# Supplementary material for: Clinical Application of the Food Compass Score: Positive Association to Mediterranean Diet Score, Health Star Rating System and an Early Eating Pattern in University Students
Source: Diseases. 2022 Jul 7;10(3):43. doi: 10.3390/diseases10030043 (PMC9326537; doi:10.3390/diseases10030043)
Supplement: Supplementary file 1 [file diseases-10-00043-s001.zip › diseases-1775080-supplementary.pdf]

**Supplementary Materials**

# Clinical Application of the Food Compass Score: Positive Association to Mediterranean Diet Score, Health Star Rating System and an Early Eating Pattern in University Students


**Paraskevi Detopoulou [1,2], Dimitra Syka [1], Konstantina Koumi [2], Vasileios Dedes [1], Konstantinos Tzirogiannis [3] and Georgios I. Panoutsopoulos [1,*]**

1 Department of Nutritional Science and Dietetics, Faculty of Health Sciences, University of Peloponnese, New Building, Antikalamos, 24100 Kalamata, Greece; viviandeto@gmail.com (P.D.); beta1967@hotmail.com (D.S.); vdedes@hotmail.com (V.D.); gpanouts@uop.gr (G.I.P.)
2 Department of Clinical Nutrition, General Hospital Korgialenio Benakio, Athanassaki 2, 11526 Athens, Greece; viviandeto@gmail.com (P.D.); linakoumi@hotmail.com (K.K.)
3 Internal Medicine Department, Mediterraneo Hospital, 16675 Athens, Greece; tzirocon@gmail.com
* Correspondence: gpanouts@uop.gr


**Supplementary Table S1: Component loadings derived using Principal Component Analysis for the identification of time- related meal patterns.**

| | *Early eaters* | *Medium eaters* | *Late eaters* |
|---|---|---|---|
| **Breakfast** | **0.726** | 0.344 | -0.341 |
| **Snack** | **0.811** | -0.037 | 0.207 |
| **Meal** | -0.007 | **0.817** | -0.078 |
| **Afternoon snack** | **0.720** | -0.063 | 0.346 |
| **Dinner** | 0.073 | **0.672** | 0.407 |
| **Bedtime snack** | 0.187 | 0.103 | **0.869** |
| **% Cumulative variance** | 33.7 | 53.07 | 70.0 |

Higher absolute values of the loadings indicate that the meal is correlated with the respective component. Numbers in bold indicate absolute loadings greater than 0.45.

**Supplementary Table S2: Pearson partial correlation coefficients between ranked scores and ranked meal patterns adjusted for age, sex, BMI and underreporting.**

| *Ranked variables of scores/ meal patterns* | | **FCS** | **HSR** | **MedDietScore** |
|---|---|---|---|---|
| **Early eaters** | rho | **0.207** | **0.178** | **0.186** |
| | p | **<0.001** | **0.001** | **0.001** |
| **Medium eaters** | rho | 0.047 | 0.020 | 0.084 |
| | p | 0.396 | 0.723 | 0.130 |
| **Late eaters** | rho | -0.104 | -0.071 | **-0.125** |
| | p | 0.059 | 0.198 | **0.024** |

Significant correlations are shown in bold.

FCS: Food Compass Score; HSR: Health Star Rating; MedDietScore: Mediterranean Diet Score
